# Therapeutic Contribution of Tau-Binding Thiazoloflavonoid Hybrid Derivatives Against Glioblastoma Using Pharmacological Approach in 3D Spheroids

**DOI:** 10.3390/ijms252111785

**Published:** 2024-11-02

**Authors:** Emmanuelle T. Relave, Rayane Hedna, Attilio Di Maio, François Devred, Hervé Kovacic, Maxime Robin, Gilles Breuzard

**Affiliations:** 1 Faculté des Sciences Médicales et Paramédicales, Institut de Neurophysiopathologie (INP), UMR 7051, CNRS, Aix Marseille Université, 13005 Marseille, France; emmanuelle.relave@gmail.com (E.T.R.); rayane.hedna@uha.fr (R.H.); francois.devred@univ-amu.fr (F.D.); herve.kovacic@univ-amu.fr (H.K.); 2 Mediterranean Institute of Marine and Terrestrial Biodiversity and Ecology, IRD, CNRS UMR7263, Aix-Marseille Université, 13013 Marseille, France; attilio.di-maio@etu.univ-amu.fr (A.D.M.); maxime.robin@univ-amu.fr (M.R.); 3 Mediterranean Institute of Marine and Terrestrial Biodiversity and Ecology, IRD, NRS UMR7263, Avignon University, 84029 Avignon, France

**Keywords:** thiazole, flavonoid, microtubule-associated protein Tau, spheroid, glioblastoma, pharmacology

## Abstract

Growing evidence has unveiled the pathological significance of Tau in many cancers, including the most aggressive and lethal brain tumor glioblastoma multiform (GBM). In this regard, we have recently examined the structure–activity relationship of a new series of seventeen 2-aminothiazole-fused to flavonoid hybrid compounds (TZF) on Tau-overexpressing GBM cells. Here, we evaluated the anticancer activities of the two lead compounds **2** and **9** using multi-cellular spheroids (MCSs) which represent an easy 3D human cell model to mimic GBM organization, physical constraints and drug penetration. The two compounds reduced cell evasion from spheroids up to three times, especially for Tau-expressing cells. As a first step towards a therapeutic approach, we quantified the effects of these compounds on MCS growth using two complementary protocols: single and repeated treatments. A single injection with compound **9** slowed down the growth of MCSs formed with U87 shCTRL cells by 40% at 10 µM. More interestingly, multiple treatment with compound **9** slowed the growth of U87 shCTRL spheroids by 40% at a concentration of 5 µM, supporting the increased bioavailability of compound **9** within MCSs. In conclusion, compound **9** deserves particular attention as promising candidate for specifically targeting Tau-expressing cancers such as GBM.

## 1. Introduction

In eukaryotic cells, the Tau protein is one of the most prominent stabilizers of microtubule (MT) dynamics [1,2]. In adults, the alternative splicing of mRNA of a single gene *MAPT* can generate six predominant Tau isoforms, each with three or four MT-binding repeats located in the *C*-terminal half of the protein, and with zero to two inserts located in the N-terminal region [3]. The Tau protein has been extensively studied in neurodegenerative pathologies (so called tauopathies), notably with diverse possible post-translation modifications such as phosphorylation leading to defective microtubules and neuron functions [4,5,6]. Subsequently, growing evidence has unveiled the role of Tau expression in tumorigenesis, including in breast cancer [7,8], gastric cancer [9], prostate cancer [10,11], sarcoma [12], non-small cell lung cancer [13] and recently in brain cancers such as glioblastoma (GBM) [14,15,16,17]. Given the complicated nature of cancer progression, it would not be surprising that there exists a complex array of mechanisms involving the Tau protein where pharmacological intervention would allow opportunities to treat the disease.

**Figure 1:**
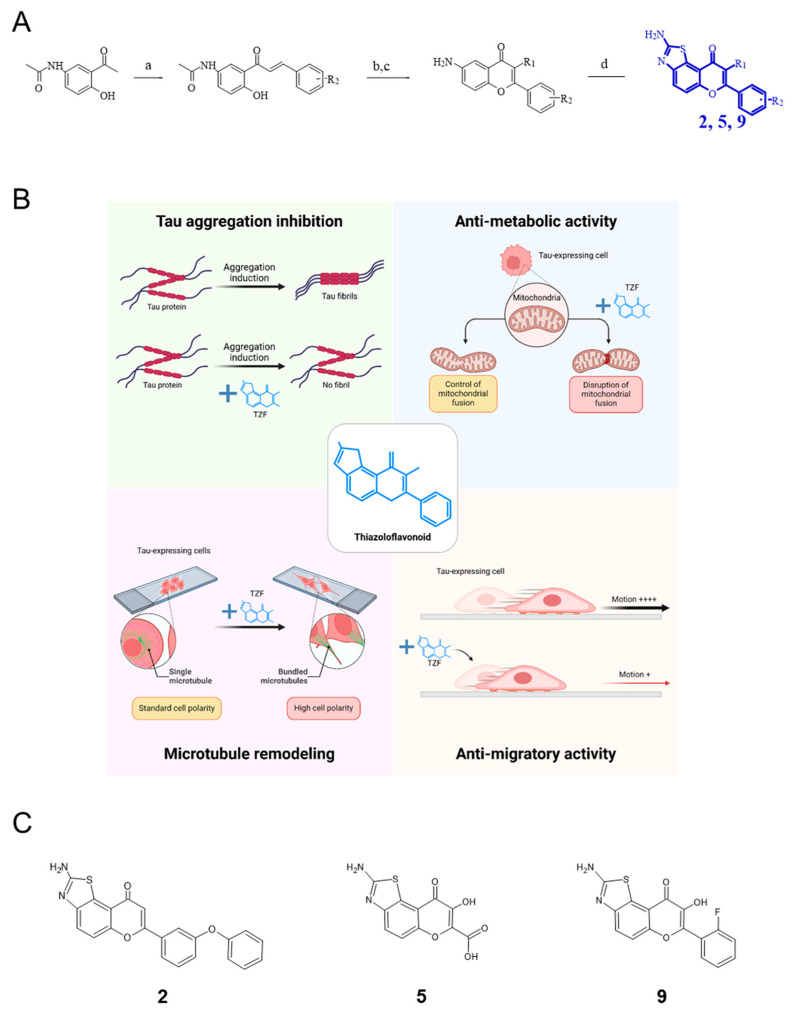
Design, synthesis and structure–activity relationship of a new series of 2-amino-thiazoloflavonoid derivatives (TZF, in blue). (**A**) Synthesis of compounds **2**, **5** and **9** (see [20]); reagents and conditions: (a) aldehyde, MeOH, LiOH, MWI (140 °C, 300 W, 20 min), (b) R2 = H, DMSO/I2 MWI (120°C, 300 W, 20 min), (c) R2 = OH, NaOH/H_2_O_2_, r.t., 24 h, (d) KSCN, AcOH, Br2, r.t., 2 h. (**B**) The structure–activity relationship of seventeen TZF derivatives was examined on Tau fibrillation and cellular effects on Tau-expressing GBM-like U87 cells; upper left panel: among tested derivatives, the two compounds **2** and **9** demonstrated high affinity for Tau and exhibited a strong propensity to inhibit Tau fibrillation; upper right panel: the two lead compounds displayed a high anti-metabolic activity on cells related to an increased fission of mitochondria network; at the bottom: both compounds induced microtubule bundling within newly formed neurite-like protrusions (left panel), as well as with defection of cell migration (right panel); created in BioRender. Relave, E. (2024) BioRender.com/y25u989, accessed on 18 September 2024. (**C**) Structures of the three analogues investigated here: concentrations half inhibiting metabolic glioblastoma-like U87 cell activity (IC50) are 1.9 ± 0.7 µM (compound **2**), 2.4 ± 1.7 µM (compound **9**) and >100 µM (compound **5**) [20].

In this context, we have recently designed, synthesized and characterized several patented flavonoid derivatives with a 2-amino-thiazole chemical group, so called thiazoloflavonoid (TZF; see patent WO2016083490A1). Therefore, we conducted first-step experiments in which the biological effects of seventeen TZF derivatives have been examined in the GBM-like U87 cell line. In addition to carrying two tumor suppressor mutations in PTEN and NF1 proteins, U87 cells exhibit an *hTERT* promoter mutation and *EGFR* gene amplification [18]. These genetic characteristics have been retained as diagnostic criteria for GBM according to the recent 2021 WHO classification of CNS tumors [19]. Furthermore, the tumorigenicity of U87 cells is significant making them an ever-interesting cell model to reproduce the GBM context. Among the TFZ derivatives tested, the two compounds **2** and **9** drew our attention due to their strong anti-proliferative, anti-metabolic and anti-migratory activities exclusively in U87 cells expressing the Tau protein (Figure 1). These observations were validated in four other GBM-derived cell lines (U118, U251, U138 and T98G) exhibiting varying levels of Tau protein expression, as well as in highly Tau-expressing neuroblastoma cells (SK-N-SH) and human differentiated epithelial cells which do not express Tau (Caco-2) [20]. Altogether, our data revealed the potential of Tau-targeting TZFs for the treatment of GBM characterized by Tau overexpression. However, our results were obtained from cells organized in a two-dimensional monolayer, which appears to be an inappropriate model for reproducing the characteristics of GBM tumorigenicity.

In the present study, we evaluated the anticancer activities of the lead compounds **2** and **9** using multi-cellular spheroids (MCSs). Indeed, MCSs can represent an easy 3D human cell model to mimic GBM organization, physical constraints and drug permeation. Our findings revealed that the two compounds were able to hinder cell evasion from spheroids, especially for Tau-expressing cells. As a first step towards a therapeutic approach, we quantified the effects of these compounds on MCS growth using two complementary protocols: single and repeated treatments. For both protocols, we measured a greater anti-proliferative effect of compound **9** on MCSs expressing the Tau protein. As expected, repeated treatment with this compound led to half the dose needed for an anti-proliferative effect equivalent to a single dose of compound, suggesting a significantly increased bioavailability of the compound within the spheroids. Taken together, these results show that compound **9** deserves particular attention as promising candidate for specifically targeting Tau-expressing cancers such as GBM.

## 2. Results

### 2.1. Compounds 2 and 9 Hinder Evasion of Tau-Expressing Cells from MCSs

**Figure 2:**
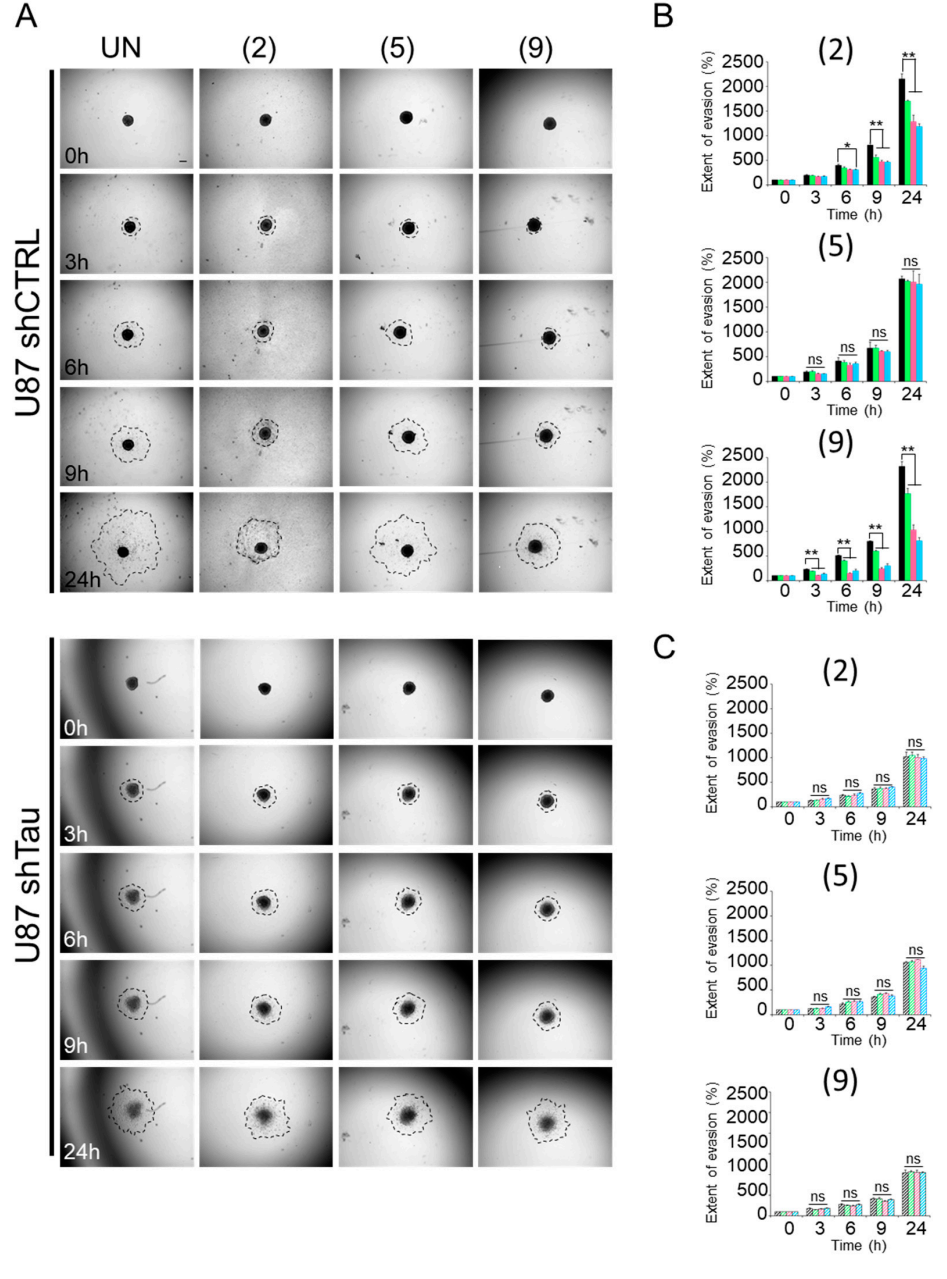
Compounds **2** and **9** hinder Tau-dependent MCS evasion. Spheroids were set on a fibronectin matrix and cell evasion analyzed by time-lapse video microscopy (Obj. ×2; at 37 °C, 5% CO_2_). (**A**) Representative images of MCSs formed with Tau-expressing U87 shCTRL (upper panel) or Tau-depleted U87 shTau (bottom panel) cells were recorded at 0–24 h of treatment with 50 µM of compounds **2**, **5** or **9**; ‘UN’: untreated; scale bar: 250 µm. (**B**) Quantification of the extent of U87 shCTRL MCS evasion incubated with 0 µM (black bars), 5 µM (green bars), 20 µM (pink bars) or 50 µM (blue bars) of compounds. (**C**) Quantification of the extent of U87 shTau MCS evasion incubated with 0 µM (hatched black bars), 5 µM (hatched green bars), 20 µM (hatched pink bars) or 50 µM (hatched blue bars) of compounds. For (**B**,**C**), all data are expressed as the mean ± SD of *n* = 9 spheroids and are representative of three independent experiments; results are expressed as the ratio of the area occupied by the migrating cells from the spheroid on the area of the spheroid at time 0 h; * *p* < 0.05, ** *p* < 0.01; ns, not significant (Bonferroni and Holm test).

Multi-cellular spheroids are proving to be valuable and beneficial tools in biomedical research, particularly for the in vitro evaluation of anticancer candidates [21,22]. They offer the possibility of testing the efficacy of existing drugs and screening new drug candidates, while providing a closer representation of the in vivo situation. By investigating the role of Tau in cells organized in spheroids, we evidenced that the protein promotes cell evasion by impacting not only cell migration but also spheroid cohesion [17]. Moreover, we recently demonstrated in a 2D cell culture model that compounds **2** and **9** induced microtubule remodeling in bundles in U87 shCTRL cells, concomitantly with the impairment of cell migration [20]. Therefore, the effect of compound concentrations on migration was evaluated on cells using a 3D MCS system. To this end, MCSs were plated on a fibronectin-coated surface, and cell evasion was monitored by time-lapse videomicroscopy (Figure 2).

As results, the two U87 shCTRL and U87 shTau cells migrated away from pre-formed spheroids over time. However, as early as 3 h, the extent of cell evasion from U87 shTau MCSs was 1.5-fold lower than that from U87 shCTRL spheroids (Figure 2A–C for quantification). The area covered by evading cells was 230 ± 7% of the area at time 0 h (t0) for U87 shCTRL, while it was 175 ± 17% for U87 shTau (*p* < 0.05). This difference was even greater at later time points (at 24 h: 2319 ± 100% of the area at t0 for U87 shCTRL vs. 1042 ± 65% of the area at t0 for U87 shTau, *p* < 0.01), indicating that U87 shTau cells migrated less from the spheroid core compared to U87 shCTRL cells. Note that U87 shCTRL core MCSs did not change in size and morphology over time, whereas U87 shTau core spheroids disassembled after 6–9 h (Figure 2A). All of these observations remain in agreement with our previous data [17].

Next, we evaluated if a treatment with compounds could affect evasion of the two cell types. To this end, we treated spheroids with increasing concentrations (5 µM, 20 µM, 50 µM) of compounds, and we measured their apparent occupied area, as before (Figure 2A–C). Treatment with compounds **2** and **9** significantly reduced U87 shCTRL cell evasion in a dose-dependent manner, up to recapitulating the phenotype of untreated Tau-depleted U87 shTau spheroids (a 1.8-times to 3-times reduction for compounds **2** and **9** at 50 μM, respectively) (Figure 2B). Our data showed that the compounds **2** and **9** were effective in hindering cell evasion from U87 shCTRL spheroids. Moreover, none of the treatments significantly affected the cell evasion from U87 shTau spheroids (Figure 2C). As expected, U87 shCTRL and U87 shTau cells evaded in the same proportion when treated with compound **5** compared to the untreated condition.

As we previously observed, the Tau protein promotes cell evasion by impacting on both cell migration and spheroid cohesion. More interestingly, compounds **2** and **9** affect the escape of Tau-expressing U87 shCTRL cells from MCSs in a dose-dependent manner. Our findings remain consistent with the profound remodeling of microtubule and actin cytoskeletons highlighted in [20]. The data strongly suggest hyperstabilization of microtubules that may originate in an accumulation of Tau along microtubules. The next step was to examine the impact of the compounds on the growth of MCSs.

### 2.2. Compound 9 Affects the Growth of MCSs

In our pharmacological approach, we monitored MCS growth to examine the antitumor effects of selected biologically active compounds **2** and **9** on three-dimensional (3D) culture models of U87 shCTRL and U87 shTau cells. The main objective was to determine how these compounds could influence tumor cell growth in an environment simulating the 3D organization of a microtumor in vivo. For this, we performed two treatment protocols: 1/ a single treatment with compounds at different concentrations deposited at the start of growth monitoring, and 2/ multiple treatments with compounds at different concentrations over time (one dose every 3 days). These results are a first step towards more in-depth pre-clinical testing.

#### 2.2.1. Protocol 1: Impact of a Single-Compound Treatment on MCS Growth

**Figure 3:**
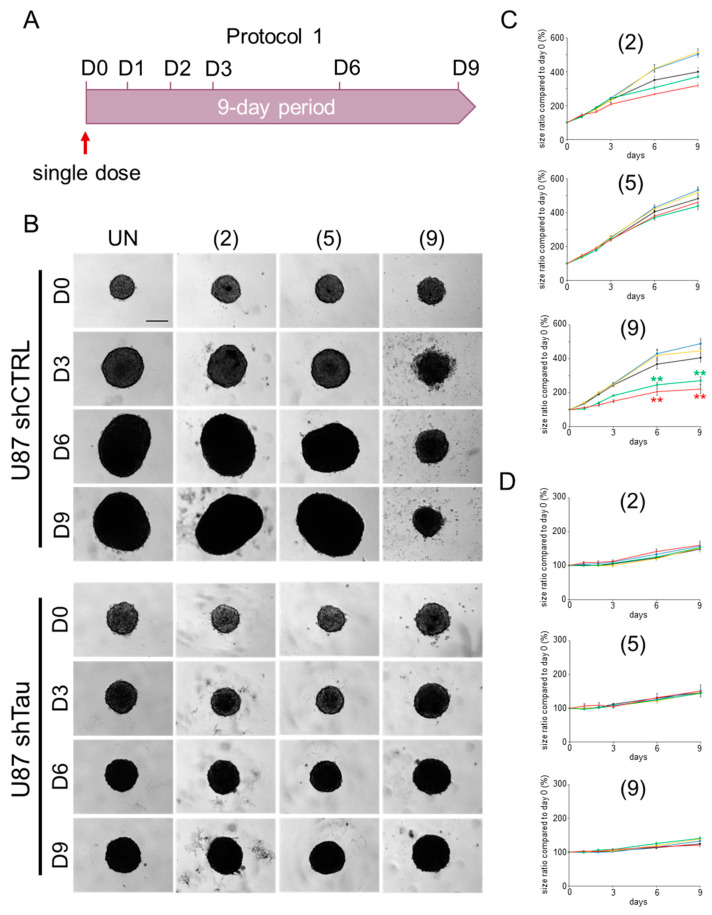
A single treatment with compound **9** reduces Tau-dependent MCS growth. A single dose of 10 µM of compound **9** reduces by 40% the growth of spheroids formed with Tau-expressing U87 shCTRL cells. The other compounds **2** and **5** have no effect at the concentrations tested. The U87 shTau cells do not respond to all of treatments. (**A**) Timeline of growth monitoring of spheroids treated with a single dose of compounds **2**, **5** and **9** over a 9-day period. (**B**) MCSs formed in the presence of methylcellulose were treated with 50 µM of compounds once on day 0 (D0), then imaged by videomicroscopy (Obj. ×4; at 37 °C, 5% CO_2_) from D0 to D9; scale bar: 250 µm. (C–D) Each graph represents the mean size of n = 9 MCSs formed with U87 shCTRL cells (**C**) and U87 shTau cells (**D**) treated with 0 µM (in black), 1 µM (in yellow), 5 µM (in blue), 10 µM (in green) and 50 µM (in red) of compounds; results are from three independent experiments ± SD. Asterisks indicate a significant difference between conditions treated (in color) and not treated (in black), with ** *p* < 0.01 (Tukey–Kramer HSD test).

A first step was to compare the impact of Tau protein expression on MCS growth. The results are shown in Figure 3. For the no-treatment condition (black curve in Figure 3C, and ‘UN’ condition in Figure 3B, upper set of images), U87 shCTRL cells showed a 3.5-fold increase in MCS size between days D0 and D9, and the MCS appeared larger and more compact at D6. For U87 shTau cells, we observed slow but continuous growth up to 1.5 times their initial size at D9 (Figure 3D). Moreover, the MCSs also appeared more compact from D6 (Figure 3B, bottom set of images). Our data are in agreement with previous observations that Tau expression greatly promotes MCS growth [17].

Moreover, we examined the impact on MCS growth of a single treatment at D0 with compounds **2** and **9** at many concentrations (0–50 µM). As for the evasion experiments above, the compound **5** served as a negative control in our data. As results, we observed that following a single treatment with the compound **9**, no significant change in the growth of MCSs formed with U87 shCTRL cells was measured for the 1 µM and 5 µM treatment doses (Figure 3C, blue and yellow curves, respectively). It is likely that these concentrations were not sufficient. In addition, for concentrations of 10 µM (green curve) and 50 µM (red curve) of compound **9**, we were able to demonstrate a reduction in MCS growth by a factor of 2, starting from growth monitoring. On the other hand, no impact on spheroid growth was determined for compounds **2** and **5** at these same concentrations. Furthermore, all three compounds had no effect on the growth of MCSs formed from U87 shTau cells (Figure 3D).

Overall, our first pharmacological approach measuring the biological effect of three compounds on a MCS model highlighted a compound of interest, compound 9, with an effect on cell growth associated with Tau protein expression. For this compound, we show growth inhibition efficacy at relatively high concentrations (10 µM and 50 µM). The next step will be to measure spheroid growth following repeated treatment with these compounds.

#### 2.2.2. Protocol 2: Impact of a Repeated Compound Treatment on MCS Growth

**Figure 4:**
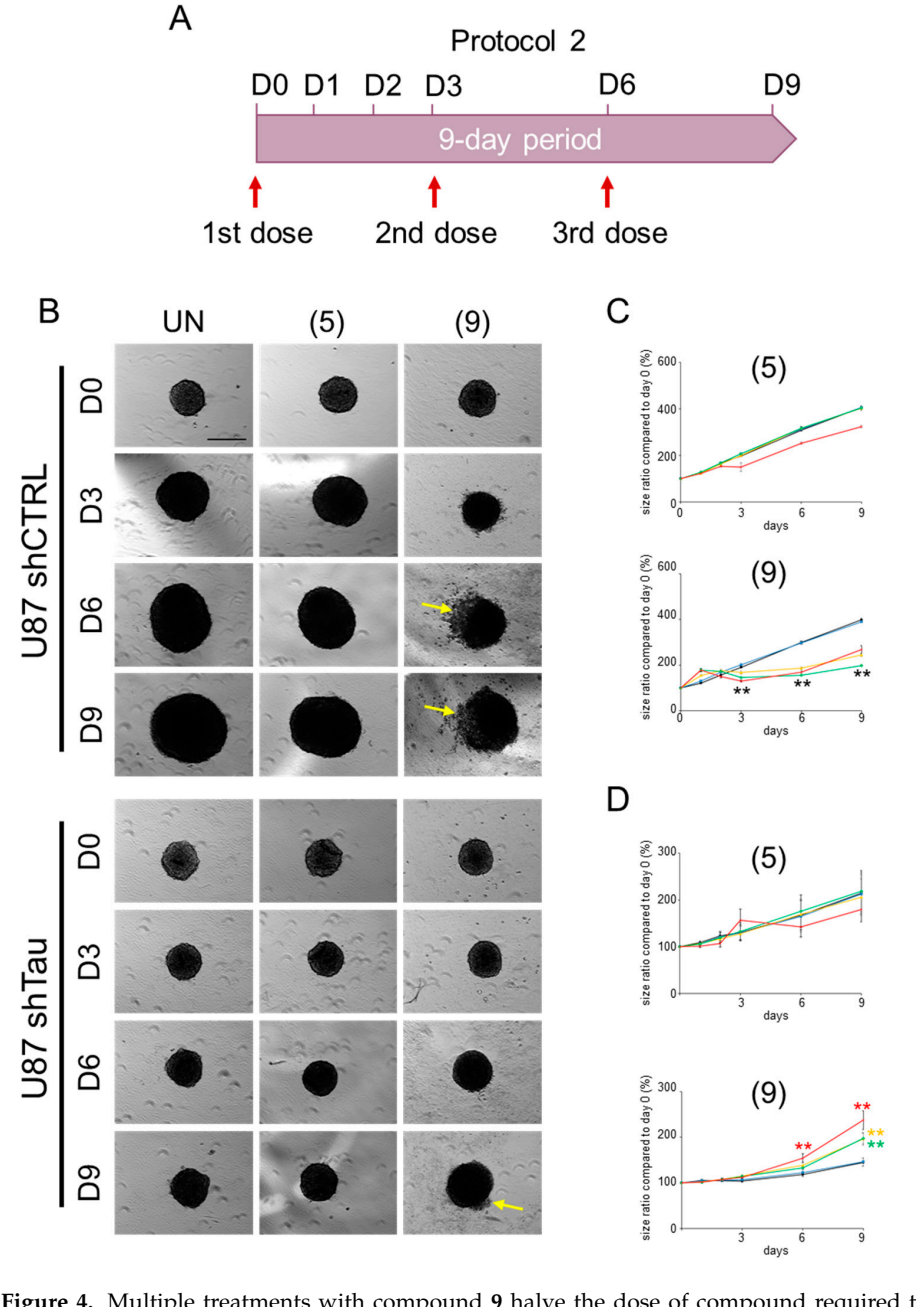
Multiple treatments with compound **9** halve the dose of compound required to slow Tau-dependent MCS growth. A repeated dose of 5 µM of compound **9** every 3 days reduces by 40% the growth of spheroids formed from Tau-expressing U87 shCTRL cells; yellow arrows show a loss of cell–cell adhesion on the periphery of MCSs in the presence of compound **9**. Compound **5** has no effect at the concentrations tested. The U87 shTau cells do not respond to all of the treatments. (**A**) Timeline of growth monitoring of spheroids treated with three doses of compounds **5** and **9** over a 9-day period. (**B**) MCSs formed in the presence of methylcellulose were treated with 50 µM of compounds every 3 days from day 0 (D0), then imaged by video microscopy (Obj. ×4; at 37°C, 5% CO_2_) from D0 to D9; scale bar: 250 µm. (**C**,**D**) Each graph represents the mean size of *n* = 9 MCSs formed with U87 shCTRL cells (**C**) and U87 shTau cells (**D**) treated with 0 µM (in black), 1 µM (in yellow), 5 µM (in blue), 10 µM (in green) and 50 µM (in red) of compounds; results are from three independent experiments ± SD. Asterisks indicate a significant difference between conditions treated (in color) and not treated (in black), with ** *p* < 0.01 (Tukey–Kramer HSD test).

The aim of this protocol was to determine the effect of compound **9** on the growth of MCSs following repeated treatment every three days with increasing concentrations (0–50 µM) of TZF. As with protocol 1, the results are presented in the form of graphs. They show the quantification of this effect on MCS growth (Figure 4C,D), as representative images of our observations (Figure 4B).

Following repeated treatment with compound **9**, we were able to demonstrate a 2-fold reduction in the growth of spheroids formed with U87 shCTRL cells, with 5 µM of **9** (yellow curve, Figure 4C, bottom graph). Unexpectedly, higher concentrations of **9** do not have a more significant effect on MCS growth. Indeed, these spheroids reached an apparent surface area comparable to the 5 µM condition. However, we could see on images recorded for 50 µM of **9** that the periphery of these MCSs showed low compactness with cells less adherent to each other as of D3 (Figure 4B, upper right panel), indicating significant cytotoxicity of compound at this concentration. Moreover, for MCSs formed with U87 shTau cells, we measured a 1.4-fold, 1.6-fold and 2-fold increase of the apparent surface area of the spheroids when treated with 5 µM, 10 µM or 50 µM of compound **9**, respectively. By analyzing the images for the 50 µM condition, we can also see a less sharp outlines of the MCSs at D9, revealing less compact spheroids at this concentration of **9** (Figure 4B, bottom right panel). These data also suggested that cell–cell adhesion became weaker at the periphery of the MCSs. Besides, we notice that repeated treatment of spheroids formed from U87 shCTRL cells and U87 shTau cells with compound **5** produced no significant response at every concentration.

Our second pharmacological approach, evaluating the potential benefits of repeated treatment with TZF compounds in a MCS model, demonstrated that repeated treatment significantly reduced the concentration of compound **9** from 10 µM to 5 µM, half the level observed in the first single-treatment protocol. This result indicated that repeated treatment increases the compound’s bioavailability within the spheroids. Our data reinforce the idea that a multiple treatment approach may be more promising than a single dose of compound **9**.

## 3. Discussion

The discovery of the Tau protein and its crucial role in tubulin assembly dates back to the 1970s [1,23,24]. Since then, our understanding of its physiological functions and pathological implications has evolved considerably. Indeed, Tau has emerged as a multifunctional protein, whose role extends far beyond the regulation of microtubule dynamics [25]. Dysregulation or alteration of Tau expression can be associated with a variety of pathologies, including neurodegenerative diseases and, more recently, certain types of cancer. The development of new agents targeting Tau remains an active area of research. As such, our main objective was to present new drug candidates targeting Tau with strong therapeutic potential. Given the interactions demonstrated by flavonoids with the Tau protein, we have opted for thiazoloflavonoid hybrid derivatives, some molecules in which a thiazole group is integrated into the flavonoid heterocycle, as our initial candidates, and we have demonstrated their therapeutic potential in a cancer model overexpressing Tau protein, such as GBM [26,27,28].

Encouraged by the promising results obtained in 2D cell models [20], we decided to take our analysis a step further by assessing the impact of the more active compounds **2** and **9** on both Tau-dependent migration and the growth of MCSs in 3D. The MCS model does not fully capture the GBM ecosystem in brain tissue, as it lacks the diversity of cell types (neurons, immune cells, endothelial cells, blood cells, microglia), chemical factors (hormones, chemokines, cytokines, etc.) and physical factors (mechanics, dissolved gases, blood–brain barrier). Nevertheless, this in vitro approach is a first step towards preclinical experiments, aiming to better represent the in vivo environment and offer a more global perspective on compound efficacy, taking into account the complexity of cellular interactions in a 3D context. It would therefore be important to validate all the results observed in an animal model (such as the mouse) more suitable for reproducing the characteristics of GBM, while considering cellular and molecular heterogeneities, differences in accessibility to nutrients as well as O_2_ and pH variations if necessary (see the well-detailed review [29]).

Regarding data about cell migration, we confirmed that Tau expression in U87 shCTRL cells promoted cell evasion from MCSs, as previously observed [17]. Interestingly, a treatment with compounds **2** and **9** halved the distances covered by U87 shCTRL cells expressing Tau, whereas U87 shTau cells depleted for Tau were not affected. These observations remain in agreement with our previous data in which compounds **2** and **9** induced a profound remodeling of microtubule cytoskeleton in cells expressing Tau [20]. Using in vitro turbidimetry, we previously excluded the possibility of an influence of the compounds on the rate of Tau-mediated polymerization of microtubules, suggesting a potent accumulation of Tau on microtubules leading to the stabilization and the remodeling of the cytoskeleton in cells. Besides, the interaction of these compounds with Tau could protect the protein from hyperphosphorylation mediated by various kinases. The dysregulation of Cyclin-dependent kinase 5 (Cdk5) and Glycogen synthase kinase-3β (GSK3β) has been associated with both neurodegenerative disorders and multiple cancers [30,31,32,33]. Tau can simultaneously bind to Cdk5 and GSK3β, forming a complex in which Cdk5 phosphorylates Tau at serine 202 and threonine 205,priming it for subsequent phosphorylation at serine 199 by GSK3β. Similarly, Cdk5 primes Tau for sequential phosphorylation at serine 400 and 396 by GSK3β through initial phosphorylation at serine 404. Interestingly, tyrosine 197 and 304 are close to these phosphorylated sites on Tau. Using a spectrofluorometric approach (see [20]), we determined the binding constants of TZF derivatives to Tau from the quenching of the fluorescence of five tyrosine residues (tyrosine 8, tyrosine 29, tyrosine 197, tyrosine 310 and tyrosine 394). Indeed, the Tau fluorescence emission gradually decreased with increasing concentrations of compounds, indicating that these small molecules interact with the tyrosine-residues of Tau near the binding site. Therefore, the binding of compounds **2** and in particular **9** to these tyrosine-containing sites on Tau could induce steric hindrance for kinases, resulting in increased interaction of Tau with microtubules, its main ligand.

To investigate the anti-proliferative activity of this new family of compounds, we compared two pharmacological protocols: a single vs. repeated treatment of MCSs with compounds over time. Surprisingly, our data showed in both approaches that only compound **9** reduced significantly the growth of MCSs formed with Tau-expressing U87 shCTRL cells. The biological activities of compounds **2** and **5** may be influenced by several extra- and intracellular factors, such as differences in membrane permeability between cells organized in 2D versus 3D, as well as active drug metabolism mediated by proteins like cytochromes that represent the most significant problems in clinical pharmacology (see the well-described review [34]). Our findings confirm the interest of fluorinated residue in compound 9 over the phenoxybenzene (compound **2**) or carboxylic (compound **5**) substituents on the C ring. Moreover, the anti-proliferative activity required relatively high concentrations of **9** compared with results obtained in a 2D system [20]. These observations suggest that the 3D organization of U87 shCTRL cells could act as a barrier to compound penetration to spheroids, particularly due to the expression and placement of cell adhesion proteins on the cell surface such as N-cadherin [17,35,36,37]. Furthermore, a repeated treatment with compound **9** significantly reduced the concentration required to achieve the same effect in Tau-expressing cells, suggesting an increased bioavailability of the compound within the spheroids. Similar results have been obtained in other studies comparing multiple treatments with a single one [38]. Besides, it has been clearly reported that Tau can interact with every component of the PI3K/Akt signaling pathway, including PTEN [15,17,39,40]. In this context, we recently conducted an in silico analysis of reverse phase protein arrays data from a cohort of GBM patients (TCGA-GBM dataset) [17]. We found a significantly lower level of activated Akt, especially in patients expressing low levels of Tau RNA (*MAPT* gene, with *p*-value = 0.0078). Furthermore, we have demonstrated that Tau is an upstream regulator of PI3K/Akt signaling, notably via microtubule remodeling. Mechanistically, the binding of compound **9** to Tau would stabilize the microtubule network, thereby disturbing the proper delivery of PI3K/Akt proteins, which in turn negatively affects epithelial-to-mesenchymal transition, cell survival and proliferation in GBM cells.

Moreover, the transcription factor, p53 (encoded by the *TP53* gene), is a critical broad-spectrum suppressor regulating over 2500 genes involved in tumorigenesis and tumor invasion [41]. Under normal physiological conditions, p53 promotes DNA repair, cell cycle arrest and cell apoptosis in response to diverse stress signals such as DNA damage. Thus, an inactivating mutation in *TP53* or its negative regulation contributes to tumorigenesis. Based on a multidimensional and comprehensive characterization of over 500 GBM samples, Brennan and coll. clearly identified the mutation or deletion of *TP53* in approximately 28% of cases, amplification of the p53-negative regulator Mdm2/4 in 15%, and/or deletion or mutation of its own negative regulator CDKN2A (as in U87 cell model) in 58% of patients [42]. Although a direct relationship between Tau and p53 has not yet been clearly demonstrated in GBM, Sola et al. recently showed in a neuroblastoma cell model that Tau downregulation affected p53 stability, influencing cell fate by increasing cellular senescence and decreasing apoptosis [43]. Meanwhile, it is well-established that the p53 levels are increased in the AD brain, where they are accompanied by an accumulation of hyperphosphorylated Tau (see the review [44]). Mechanistically, Tau spontaneously activates endogenous kinase(s), likely Cdk5 and GSK3β, that prevent the activating phosphorylation of p53 at Ser33, thereby protecting neurons from p53-driven pro-apoptotic elements [45]. This results in the apoptotic resistance of neurons, which in turn prolongs the neurodegenerative process over years. Altogether, it is tempting to speculate on the dual therapeutic benefit of TZF compounds: by interacting with Tau leading to microtubule remodeling, compounds **2** and particularly **9** could indirectly enhance p53 delivery to the nucleus, thereby reducing tumorigenesis; in tauopathies, our compounds could protect Tau from kinase-mediated phosphorylation and aggregation, particularly by Cdk5 and GSK3β, which also antagonize the pro-apoptotic function of p53.

## 4. Materials and Methods

### 4.1. Chemistry

The synthetic process and full characterization data of compounds **2**, **5** and **9** are completely depicted in [20].

### 4.2. Biology

#### 4.2.1. Cell Culture

The generation and characterization of the U87 shCTRL and U87 shTau2 cell clones by the shRNA approach were previously described in [16]. Cells were continuously maintained by regular passages in complete Dulbecco’s Modified Eagle Medium (DMEM, Lonza, Basel, Switzerland) supplemented with 10% fetal bovine serum (FBS, Lonza), 2 mM of L-glutamine (Invitrogen, Thermo Fisher Scientific, Illkirsh, France) and 0.4 µg/mL puromycin at 37°C in a 5% CO_2_ atmosphere.

#### 4.2.2. Multi-Cellular Spheroid (MCS) Cell Evasion Assay

Adherent cells were detached by Trypsin-EDTA 0.05% (Thermo Fisher Scientific, Waltham, MA USA), counted and resuspended in a solution of 20% methylcellulose (Sigma-aldrich, Saint-Quentin Fallavier, France) in DMEM complete medium (2.5 × 10^4^ cells per mL). Then, the cells were plated in 96-well suspension culture U bottom plates (Greiner Bio-one, Les Ullis, France) (2500 cells per well). Newly formed MCS (2500 cells) were settled on a fibronectin extracellular matrix (10 μg/mL in phosphate buffer saline, DPBS 1×). Images of evading cells from spheroids were taken by time-lapse video microscopy with a wide-field inverted microscope (obj dry 2× Eclipse TE 2000, Nikon Instruments Inc., Melville, NY, USA), provided with a motorized stage and connected with a CCD camera CoolSNAP HQ, driven by NIS elements AR software (Nikon; version 2.30). The microscope system was provided with a 37°C incubation chamber and a CO_2_ and humidity control system. One frame was taken every 3 h for 24 h. Quantification of cell evasion was performed from images taken at every time using ImageJ software (version 1.54j). The extent of cell evasion from MCS was analyzed by ImageJ software (version 1.54j) and results expressed as the following: (area of cell evasion at each time point) / (spheroid area at time 0 h); mean ± SD, *n* = 9 spheroids. The cumulated results were from four experiments.

#### 4.2.3. MCS Growth Assay

Spheroid formation and initial culture were as described for the evasion experiments. After 24 h, formed MCSs were transferred to a new U-bottom 96-well plate in 200 µL of complete culture medium supplemented with 10%FBS, and treated with different concentrations of compounds (from 0–50 µM) according to two distinct protocols: 1/ protocol 1 consisted of a single treatment on the first day of MCS growth monitoring (D0); 2/ protocol 2 consisted of repeated treatment every three days from D0 (days D0, D3, D6 and D9). MCSs received fresh medium supplemented with appropriate concentration of compounds. Spheroid growth was observed with a wide-field Eclipse TE2000 inverted microscope (Nikon Instruments Inc., Melville, NY, USA) supplied with a 4× dry objective and equipped with a CCD camera CoolSNAP HQ (Photometrics, Tucson, AZ, USA), driven by NIS elements AR software (Nikon; version 2.30). One image per spheroid was taken at days 0, 1, 2, 3, 6 and 9. The area occupied by the spheroid was measured from images by ImageJ software (version 1.54j) and expressed as the percentage of the spheroid area at day 0 (mean ± SD, *n* = 9 spheroids). The experiments were repeated at least three times.

#### 4.2.4. Statistical Analysis

Data are presented as mean ± SD. For analysis of cell evasion and MCS growth, multiple comparisons were performedusing one-way ANOVA with a Bonferroni and Holm comparison test and a post-hoc Tukey–Kramer HSD test, respectively. For all conditions, differences were considered as statistically significant with * *p* < 0.05 and ** *p* < 0.01. Asterisks in graphs indicate significant level vs control.

## 5. Conclusions

In conclusion, our study has highlighted the promising therapeutic potential of 2-aminothiazole-flavonoid hybrid derivatives in modulating the Tau protein in cancers expressing this protein. We demonstrated here that these new drugs, in particular 2-amino-7-(2-fluorophenyl)-9H-chromeno [6,5-d]thiazol-9-one (compound **9**), significantly reduced the growth of Tau-expressing spheroids, as well as cell evasion from MCSs. Our results provide proof that compound **9** deserves particular attention as a promising candidate for specifically targeting Tau-expressing cells in the context of cancer such as GBM.

## Data Availability

The data presented in this study are available on request from the corresponding author. The data are not publicly available due to patent applications pending for several of the compounds studied in this study.
